# Spike threshold dynamics reshape the phase response curve and increase the degree of synchronization among neurons coupled by excitatory synapses

**DOI:** 10.1186/1471-2202-13-S1-P12

**Published:** 2012-07-16

**Authors:** Michael A Farries, Charles J Wilson

**Affiliations:** 1 Department of Biology, University of Texas San Antonio, San Antonio, TX 78240, USA

## 

The collective behavior of a neuronal population can often be illuminated by representing neurons as simple phase oscillators, where the response of each neuron to its synaptic input is given by an infinitesimal phase response curve (iPRC). This approach can, for example, predict whether neuronal activity will tend to synchronize across the population depending on the nature of the synaptic coupling and the shape of the iPRCs[1-3]. Despite the extreme simplicity of phase models, we have found that the response of subthalamic neurons to excitatory synaptic input is remarkably well described by iPRCs; this is probably true of many other cell types. However, one aspect of subthalamic neurons’ response to input may undermine the phase model description: spike threshold accommodation[4], a phenomenon also found in many other cell types. In spike threshold accommodation, spikes fired following more rapid depolarization are triggered at a lower voltage threshold. We developed a phenomenological model of spike threshold accommodation that treats the threshold itself as a dynamical variable; this model successfully accounted for the experimentally observed features of this phenomenon[4]. However, the introduction of a new dynamical variable raises the possibility that one-dimensional phase models will be unable to represent adequately the impact of spike threshold dynamics. This danger seems more pressing given that most of the threshold accommodation phenomenon arises from the fact that spikes are initiated in the axon at some distance from the soma[5,6], a factor that cannot be accounted for by standard methods for reducing a biophysical model to a phase model. We analyzed the effect of adding our phenomenological model of spike threshold dynamics to biophysical models that are otherwise well described by phase models. We discovered that spike threshold dynamics change the shape of the iPRC and that this effect alone accounts for much of this phenomenon’s impact on the response to synaptic input. Specifically, threshold dynamics increase the input sensitivity at late input phases, causing the iPRC to have higher values than one would otherwise predict. We show numerically that this alteration of the iPRC promotes synchronization of neurons coupled by excitation, even if those neurons have type I iPRCs. In addition to reshaping the iPRC itself, spike threshold dynamics also cause a deviation from the response predicted by the iPRC as the size of the stimulus grows; this effect further enhances the sensitivity to excitation at late input phases but suppresses the sensitivity to inhibition. We compare the results obtained with our phenomenological model of threshold dynamics to experimental data and to a full multicompartment biophysical model that exhibits spike threshold accommodation naturally, by virtue of the cable properties of the axon. We were able to explain some otherwise anomalous aspects of our data and predicted changes in iPRC shape as neurons are driven to fire at higher rates by DC current injection. We confirmed this effect experimentally in subthalamic neurons; a similar phenomenon has also been reported in cerebellar Purkinje neurons [7].
